# 493. ENDURANCE - EffectiveNess of Evusheld for COVID-19 Pre-exposUre PRophylAxis iN ImmunoCompromised PatiEnts – A Pilot Study

**DOI:** 10.1093/ofid/ofad500.562

**Published:** 2023-11-27

**Authors:** James Polega, Heather Brooks, Rebecca Emery, Joshua D Donkin, Kevin Fitzgerald, G O R D A N A SIMEUNOVIC

**Affiliations:** Corewell Health, Grand Rapids, Michigan; Corewell Health, Grand Rapids, Michigan; Corewell Health, Grand Rapids, Michigan; Corewell Health, Grand Rapids, Michigan; Corewell Health, Grand Rapids, Michigan; Corewell Health/ Michigan State University, Grand Rapids, Michigan

## Abstract

**Background:**

In December 2021, Evusheld, a cocktail of 2 monoclonal antibodies (mAB), was granted EUA for COVID-19 pre-exposure prophylaxis (PrEP) of immunocompromised patients (Table 1). The EUA was updated June 2022 to recommend repeating a dose every 6 months for patients needing ongoing protection. However, EUA was revoked in January 2023 due to identified resistance of Omicron lineage. Clinical trials about a new mAB for PrEP are continuing. Real-world data on the effectiveness of Evusheld may help develop strategies for the administration of new mAB.

ENDURANCE is a complex retrospective study to assess Evusheld's effectiveness by evaluating and comparing treatment outcomes among 4 groups of patients classified based on the timing of treatment and the number of repeated Evusheld treatments (Figure 1). This pilot study describes treatment outcomes in the first analyzed group (gr. A) treated before Omicron development.

Table 1

**Figure:**
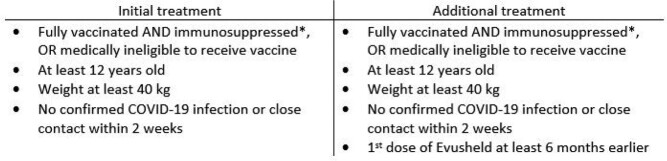

EUA criteria for Evusheld use, used as inclusion criteria for our study. * Patients with moderate to severe immunosuppression due to a medical condition or receipt of immunosuppressive medications and may not mount an adequate immune response to COVID-19 vaccination.

Figure 1

**Figure:**
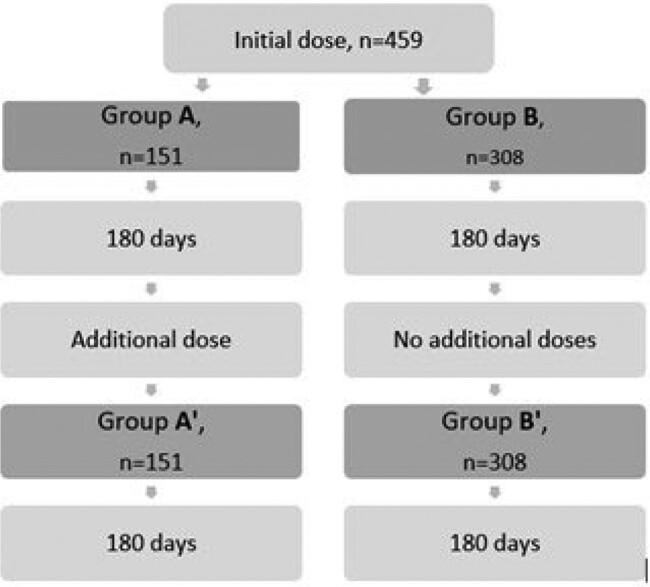

ENDURANCE Study design. 459 patients who received the initial dose of Evusheld were followed for 180 days (group A, n=151, and group B, n=308). Patients who received repeated treatment (group A’, n=151) were followed for 180 days following an additional dose while patients who did not receive repeated treatment (group B’, n=308) were followed for an additional 180 days (181 till 360 days after the initial dose). Primary outcome is defined as confirmed symptomatic COVID-19 infection. We hypothesize a significantly lower rate of COVID-19 infections in the period before Omicron emergence (Groups A and B), disregarding if patients received repeated doses of Evusheld (Group A’) or not (Group B’). A Pilot Study describes the treatment outcomes in group A.

**Methods:**

We conducted a retrospective chart review of patients treated with Evusheld under EUA between January 18 and June 21, 2022, to assess their response to treatment by evaluating outcomes listed in Figure 2. Patients were classified in tiers based on the degree of underlying immunosuppression (Table 2). In addition, COVID-19 anti-Spike antibodies were requisitioned before treatment.

Figure 2

**Figure:**
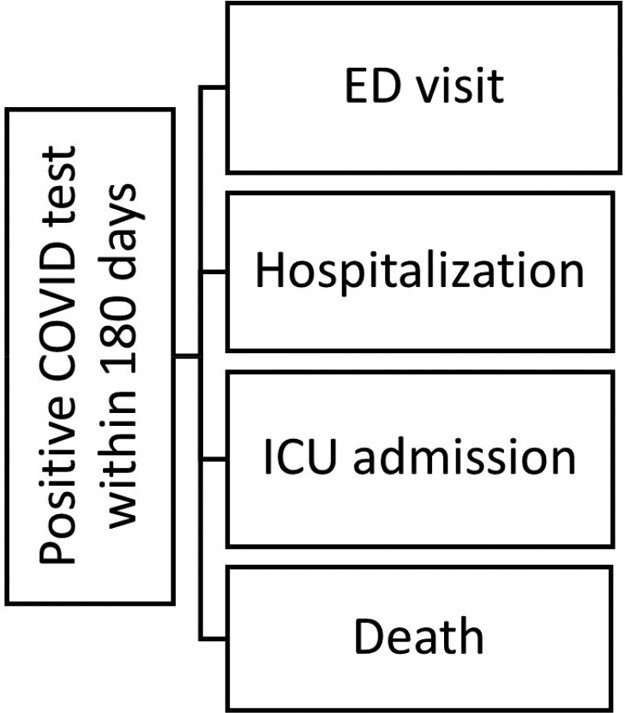

Evaluated outcomes. Primary outcome was positive COVID test at any point during the study period. Secondary outcomes were measured through the number of all-cause ED visits, hospitalizations, ICU admissions and deaths within 30 days from the positive COVID-19 test.

Table 2

**Figure:**
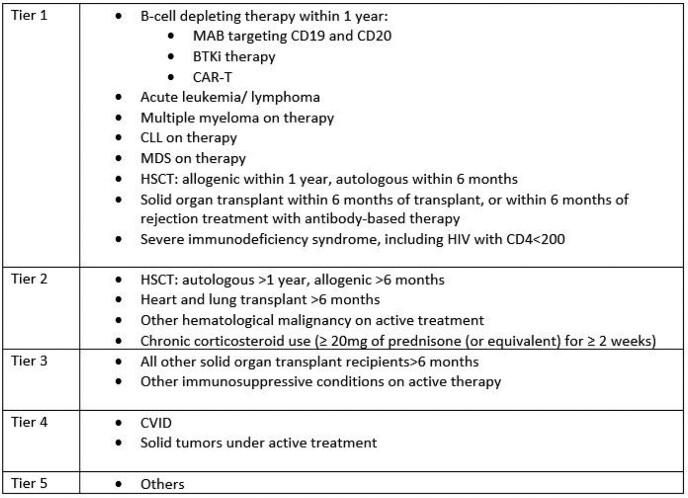

Tiered system for prioritizing patients based on severity of immunosuppression should resources be scarce.

**Results:**

From 151 patients, the majority were vaccinated (95%), seropositive (66%), and classified as Tier 1 at the time of treatment (58%) (Table 3). All seronegative patients (39) were vaccinated (Figure 3). 16/159 (11%) developed symptomatic COVID-19 infection within 180 days from treatment (mean 119.8 days); 10/16 patients received antiviral treatment outpatient (Table 4). None visited ED, were hospitalized, or died.

Table 3

**Figure:**
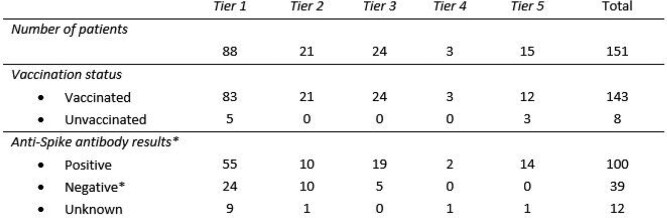

Patients’ vaccination status and anti-Spike antibody (serology) results per tiers. *All seronegative patients were vaccinated; unvaccinated patients (8) were either seropositive (5) or with unknown serology (3).

Figure 3

**Figure:**
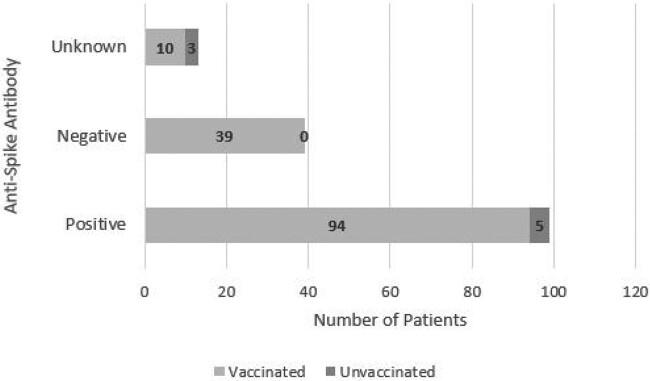

Serum anti-Spike antibody results in vaccinated and unvaccinated patients. All seronegative patients (n=39) were vaccinated. Unvaccinated patients were either seropositive (n=5) or with unknown serology results.

Table 4

**Figure:**
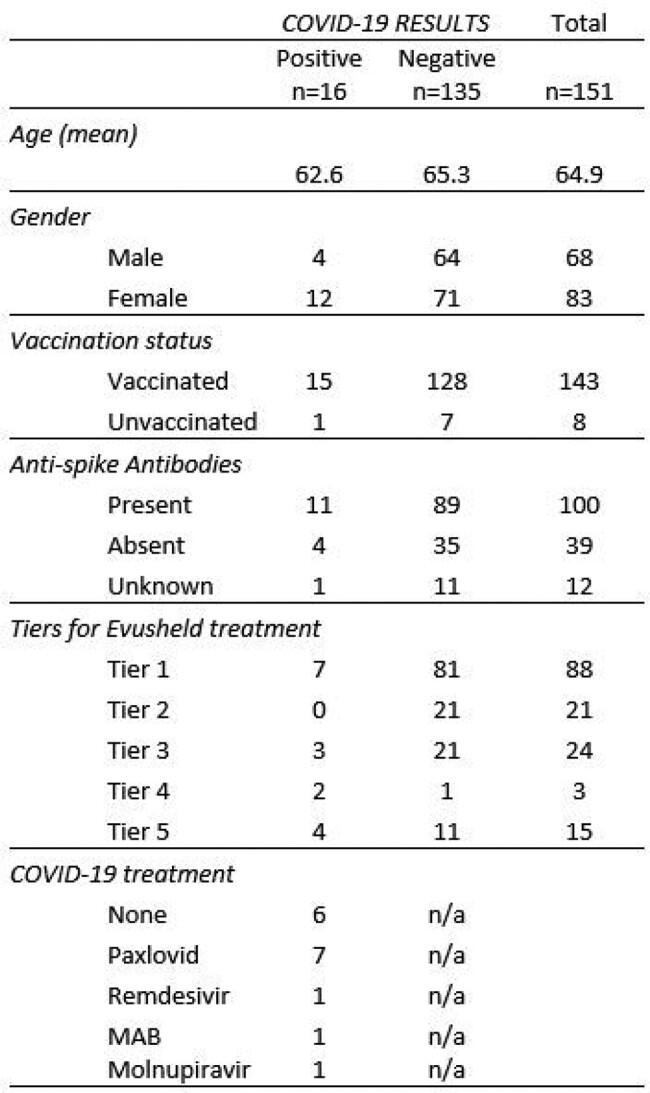

Characteristics of COVID-19 positive patients, compared to patients not diagnosed with COVID-19.

**Conclusion:**

Our pilot data suggests that Evusheld may be effective against susceptible strains, as demonstrated by the low number of symptomatic infections, which all were mild and did not require ED or hospital utilization (Figure 4). A tier system may help prioritize patients in scarce resource conditions. Further studies are needed to identify risk factors for severe COVID in immunocompromised patients and understand their immunological response to vaccines and natural infection to create the most helpful tier system.

Figure 4

**Figure:**
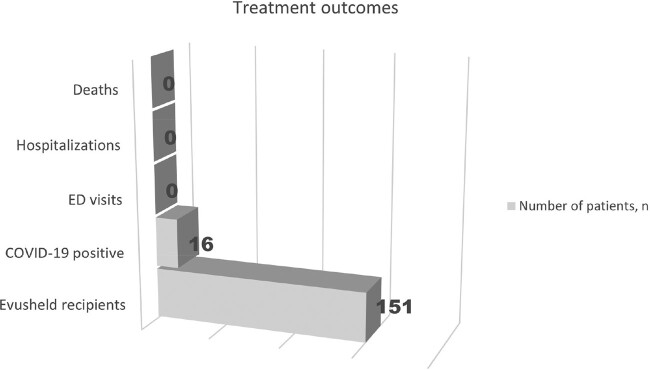

Treatment outcomes. 10% (16/151) of Evusheld recipients developed mild symptomatic COVID-19. Number of all cause ED visits, hospitalizations, and deaths within 30 days from testing positive for COVID-19 was 0, suggesting possible beneficial effect of Evusheld use in immunocompromised population.

**Disclosures:**

**All Authors**: No reported disclosures

